# Clinical outcomes of 3–5 years follow-up of immediate implant placement in posterior teeth: a prospective study

**DOI:** 10.1186/s12903-024-04058-3

**Published:** 2024-03-08

**Authors:** Rusama Wipawin, Parinya Amornsettachai, Woraphong Panyayong, Dinesh Rokaya, Sasipa Thiradilok, Patr Pujarern, Suphachai Suphangul

**Affiliations:** 1 Department of Advanced General Dentistry, Faculty of Dentistry, Mahidol University, Bangkok, 10400 Thailand; 2 Department of Prosthodontics, Faculty of Dentistry, Zarqa University, Zarqa, 13110 Jordan

**Keywords:** Dental implant, Immediate implant placement, Implant success, Implant complications, Posterior tooth

## Abstract

**Background:**

Immediate implant placement in posterior teeth has become popular in recent years. However, only a few studies focused on evaluating the long-term success of immediate implant placement.

**Purpose:**

To analyze the clinical outcomes of immediate implant placement in the posterior region with conventional loading with 3–5 years follow-up following the International Congress of Oral Implantologists (ICOI) Pisa Consensus Conference.

**Method:**

The study was done in 25 bone-level implants (Straumann® SLActive® bone level tapered implant, Straumann®, Basel, Switzerland) in 19 patients who underwent immediate implant placement in a posterior tooth with conventional loading with 3–5 years follow-up. The overall success and survival of these placements were evaluated following the International Congress of Oral Implantologists (ICOI) Pisa Consensus Conference using chart records, clinical examination, radiographic evaluation, and outcomes measurement. Patient satisfaction was evaluated by using a numeric rating scale. The biological and technical status, modified Pink Esthetic Score (mPES), complications, and marginal bone change were also evaluated. The analysis was done using SPSS version 21 (SPSS Inc., Chicago, IL, USA). The data were analyzed using a paired samples t-test.

**Results:**

It was found that 24 out of the 25 (96%) dental implants survived for an average of 57 ± 8.07 months. All of the 24 surviving dental implants were considered an operational success. The average mPES was 9.75 ± 0.44. The major prosthetic complications seen were: (1) proximal contact loss (41.67%), (2) loosening of the screw (8.33%), and (3) cement debonding (4.17%).

**Conclusions:**

Immediate implant placement in a posterior tooth with conventional loading yields a predictable result with some complications. The most prominent complications were proximal contact loss, followed by loosening of the screw and cement debonding. The implant survival rate was 96% at a mean time follow-up of 4 years and 9 months.

## Introduction

Immediate implant placement is an alternative technique to the conventional one-stage and two-stage techniques [1, 2]. Immediate implant placement minimizes bony contour and soft tissue alteration, preserves bone volume, allows for greater ease in determining the implant position for rehabilitation of the final restoration, minimizes the extent of alveolar bone loss after extraction [3–5], decreases the number of surgeries, reduces treatment time, and provides faster recovery of dental functions [6, 7]. However, case selection must be done for the immediate implant placement. The considerations for immediate implant placement are as follows: (1) medical status of the patient; (2) bony plates remaining in the socket; (3) intraradicular septum in multiple root sockets; (4) amount of apical bone; (5) diagnosis of the tooth, and (6) soft tissue health [8, 9].

Some studies were done to study the success rates of immediate implant placement. It showed that the survival rate for the immediate implant placement is approximately 95–98% at follow-up for 6 months to 2 years [10, 11]. Meijer et al. [12] reported only 73.3% survival rate of immediate placement of implants in the molar site at 1-year follow-up which shows an increased failure rate over time. Similarly, another meta-analysis [13] reported the risk of immediate implant placement failure is increased by 3% after at least a 1-year follow-up. In comparison to delayed implant placement, immediate implant placement demonstrates a lower survival rate [10]. Complications of soft tissue and marginal bone changes occur within a year following immediate implant placement [14, 15], compared to other complications [11]. Furthermore, a study found that the survival rate of immediate implant placement in posterior teeth was 96% while the success rate in molar teeth was 93% after the 1-year follow-up [16].

As there are limited studies on long-term follow-up in the posterior tooth area studying the complications and their related factors, this study aimed to analyze the clinical outcomes of immediate implant placement in the posterior region with conventional loading with 3–5 years follow-up following the International Congress of Oral Implantologists (ICOI) Pisa Consensus Conference using chart records, clinical examination, radiographic evaluation, and outcomes measurement. In addition, the biological and technical status, modified Pink Esthetic Score (mPES), complications, marginal bone change, and patient satisfaction were evaluated.

## Method

### Study design and subjects

This is a prospective study where we analyzed clinical outcomes of immediate implant placement in the posterior region with conventional loading with 3–5 years follow-up following the International Congress of Oral Implantologists (ICOI) Pisa Consensus Conference. The study protocol was approved by the Institutional Review Board of the Faculty of Dentistry/Faculty of Pharmacy, Mahidol University (MU-DT/PY-IRB 2022/006.2801) and registered with the Thai Clinical Trials Registry (TCTR20220809006). Written informed consent was obtained from all participants.

The inclusion criteria of the subjects were as follows.

- Patients requiring placement in the posterior region.
- ASA I and II patients who can undergo immediate implant placement and restorative procedures.
- Both males and females.
- Patient’s age: 33–76 years old.
- Patients who can come for the follow-up visit.
- Patients who provide written informed consent.

The exclusion criteria of the subjects were as follows.

- Pregnancy.
- Smoking: >10 cigarettes per day.
- Patients with previously failed dental implants at the implant placement site.
- Patients with active infections.
- Other medical conditions that might affect the osseointegration of dental implants, such as diabetes, cardiovascular disease, hypertension, and osteoporosis [17].

### Data collection

**Fig. 1:**
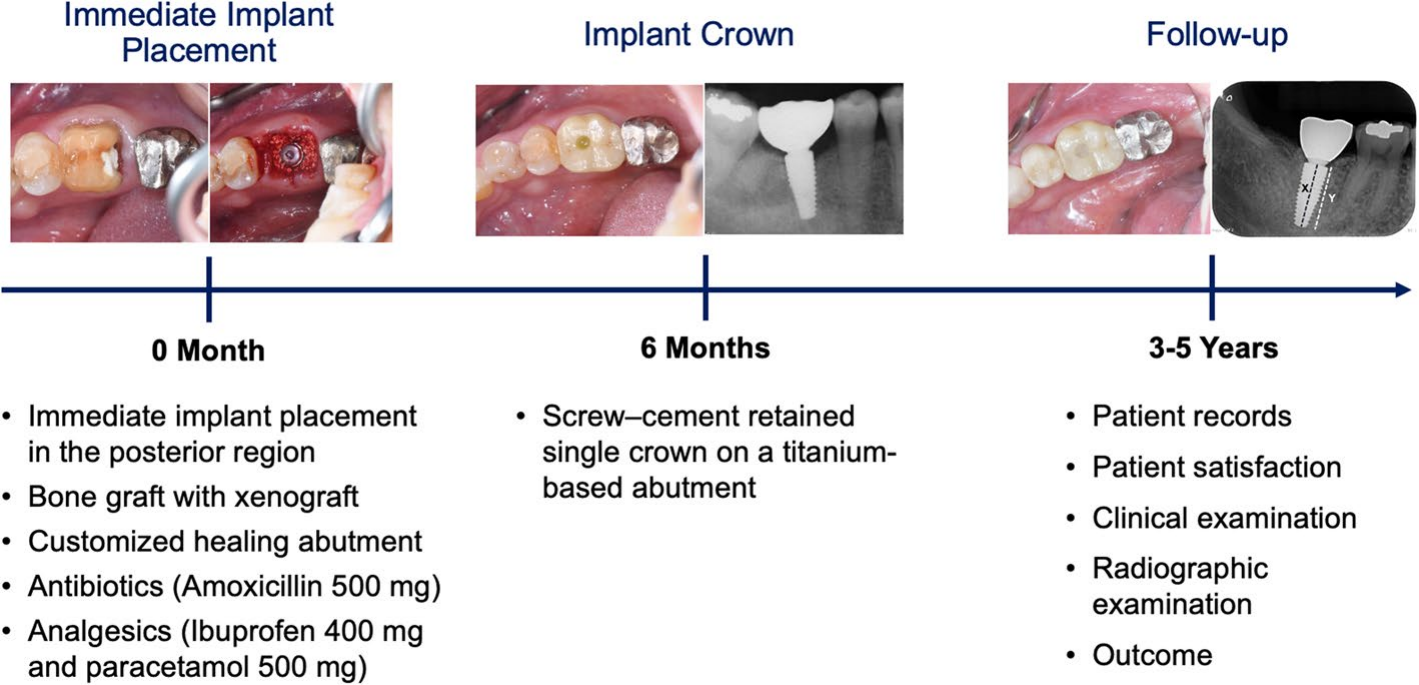
Overview of the study

This study analyzed 25 dental implants (Straumann® SLActive® bone level tapered implant, Straumann®, Basel, Switzerland) in 19 patients with immediate implant placement in the posterior region from 2016 to 2018 at the Advanced General Dentistry clinic, Mahidol University, Thailand. For all cases, xenografts (Cerabone®, botiss, Zossen, Germany) were used and covered with customized healing abutment (Variobase®, Straumann®, Basel, Switzerland and Protemp™ 4, 3M ESPE, Minnesota, USA and Filtek™ Z350 XT flowable composite, 3M ESPE, Minnesota, USA). Following the implant surgery, Amoxicillin (500 mg) was prescribed for 7 days, Ibuprofen (400 mg) was prescribed for 3 days, and Paracetamol (500 mg) as needed. At 6 months of implant placement, the implants were restored using a screw–cement retained single crown on a titanium-based abutment (Variobase®, Straumann®, Basel, Switzerland). The surgeries and restorations were performed by one oral surgeon and one prosthodontist. Details of the information were recorded from the chart records, patient satisfaction, clinical examination, radiographic evaluation, and outcomes measurement at a minimum of 3-year follow-up as shown in Fig. 1.

#### Chart records

The following information was recorded.

- Age, gender, and medical status (Table 1).
- Implant site characteristics (Table 2).
- Dental implant and restoration characteristics: implant dimensions, and material of the prosthesis (Table 3).
- Any complications.

**Table 1:** Patient demographic data

Age (years)	Mean ± SD	56 ± 15.03
	Range	33—76
Gender	Male (n, %)	6, 31.58%
	Female (n, %)	13, 68.42%
Underlying disease	Present (n, %)	10, 52.63%
	None (n, %)	9, 47.37%

*SD* Standard deviation

**Table 2:** Characteristics of dental implant sites (*n* = 25)

**Implant site characteristics**	**n, %**
Site	
Premolar	7, 28.00%
Molar	18, 72.00%
Arch	
Maxilla	10, 40.00%
Mandible	15, 60.00%
Gingival biotype	
Thick	17, 68.00%
Thin	8, 32.00%

**Table 3:** Dental implant and restoration characteristics (*n* = 25)

**Implant and restoration characteristics**	**n, %**
Implant size (diameter and length)	
4.1 × 10 mm	1, 4.00%
4.1 × 12 mm	6, 24.00%
4.8 × 10 mm	10, 40.00%
4.8 × 12 mm	8, 32.00%
Implant prosthesis	
Zirconia crown	23, 92.00%
Full metal crown	2, 8.00%

#### Patient satisfaction

The patient satisfaction for aesthetics, function, sense, speech, and self-esteem was evaluated by a numeric rating scale ranging from 0 to 5, where 0 represents “very dissatisfied” and 5 represents “very satisfied” as shown in Table 4 [12]. The overall satisfaction score was scored from 0–10, where 0 represents the least satisfied and 10 represents the maximum satisfied.

#### Clinical examination

The following information was recorded from the intraoral examination.

- Oral Hygiene Index (OHI-S) [18].
- Width of keratinized mucosa.
- Probing depth, bleeding, or suppuration on probing.
- Pain and infection.
- Mobility.
- Interproximal contacts.
- Esthetics, which were evaluated by modified Pink Esthetic Score (mPES) according to the study of Belser et al. [19] as shown in Table 4.
- Any implant and prosthetic complications.

**Table 4:** Patient satisfaction evaluation

**Patient satisfaction**	**Mean ± SD**
Aesthetic (score 0–5)	4.75 ± 0.53
Function (score 0–5)	4.67 ± 0.64
Sense (score 0–5)	4.71 ± 0.46
Speech (score 0–5)	5.00 ± 0.00
Self-esteem (score 0–5)	4.71 ± 0.46
Overall satisfaction (score 0–10)	9.25 ± 0.90

*SD* Standard deviation

#### Radiographic examination

**Fig. 2:**
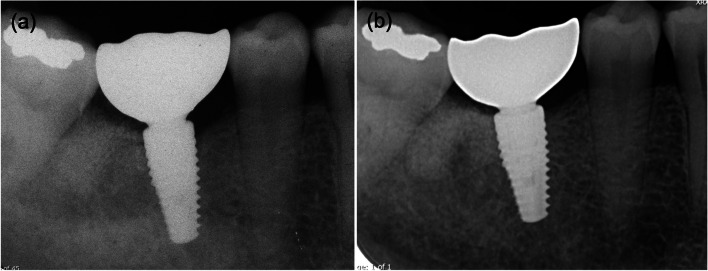
Radiographs of tooth 46: **a** post-operative radiograph after crown installation; **b** follow-up at 5 years and 6 months after implant placement

Periapical radiographs were taken using a paralleling technique as shown in Fig. 2 and the following information was recorded.

**Fig. 3:**
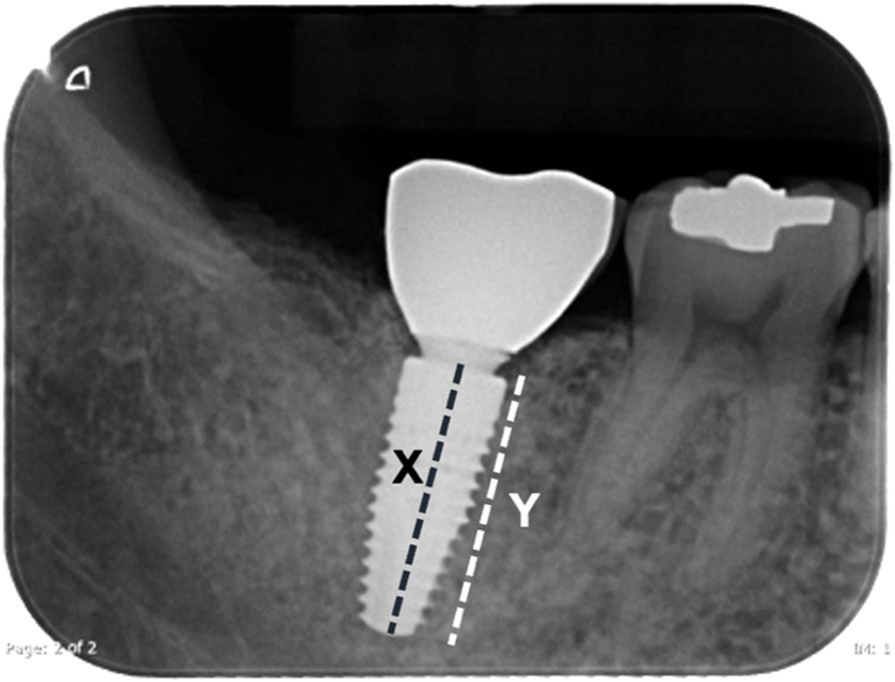
Measurement of X and Y parameters to assess marginal bone change from the periapical radiograph

- Marginal bone levels as calculated as follows [20].
  ◦ Implant length (X) was measured from the platform to the bottom margin of the implant (Fig. 3).
  ◦ Bone level (Y) was measured from the lowest margin of the marginal bone next to the implant surface to the bottom of the implant parallel to X (Fig. 3).
- Any peri-implant radiolucency.

The marginal bone changes were measured from the radiographs (DICOM files) via the picture archiving and communication system (PACS) (Version: 5.7.100, FUJIFILM Worldwide, FUJIFILM Medical Systems, Inc., North Carolina, USA) using X and Y parameters. The marginal bone changes (mm) were calculated using the formula $\frac{\mathrm{x}-\mathrm{y}}{\text{x}}\text{*R}$, where R is the length of the implant.

### Outcome measurement

**Fig. 4:**
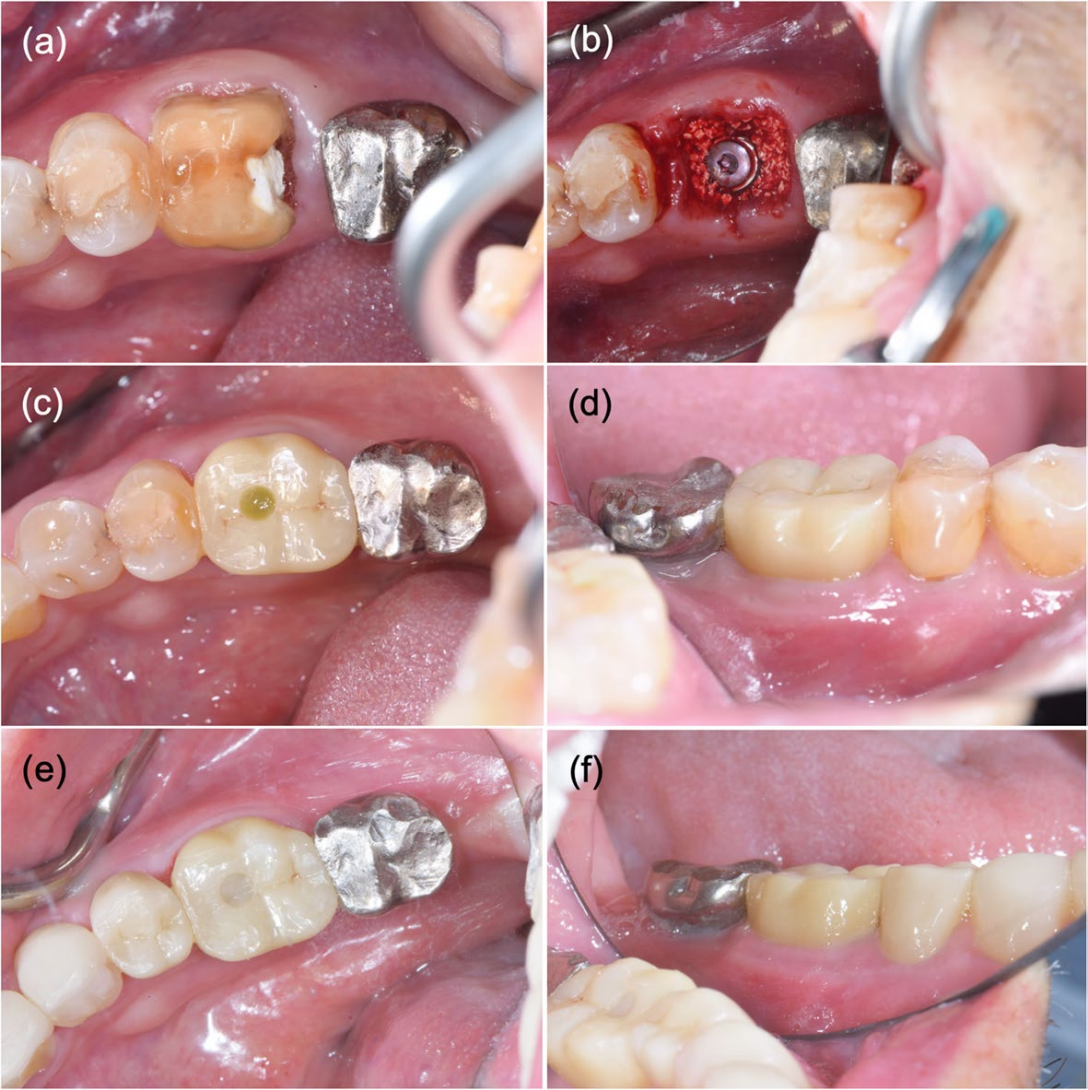
Photographs of tooth 46 immediate implant placement in posterior tooth area: **a** tooth 46 diagnosed as unrestorable tooth; **b** immediate implant placement with xenograft bone grafting; **c**, **d** screw–cement-retained zirconia crown installation 6 months after surgery (occlusal, buccal aspect); **e**, **f** follow-up 4 years and 9 months after implant placement (occlusal, buccal aspect)

Oral hygiene evaluation from the OHI-S index was calculated from the debris index (DI) and calculus index (CI) and interpreted as good (score 0-1.2), fair (score 1.3-3.0), and poor (score 3.1-6.0) [18]. The mPES was reported in the mean score and a 6 mark is required for the clinical acceptance. Photographs of surgery protocol, prosthesis installation 6 months after surgery, and prosthesis at the time of follow-up are shown in Fig. 4.

 The success and survival of dental implants were defined based on the International Congress of Oral Implantologists (ICOI) Pisa Consensus Conference as shown in Tables 5 and 6 [21]. The success of the osseointegrated implants was determined under a functional load at the time of evaluation.

**Table 5:**
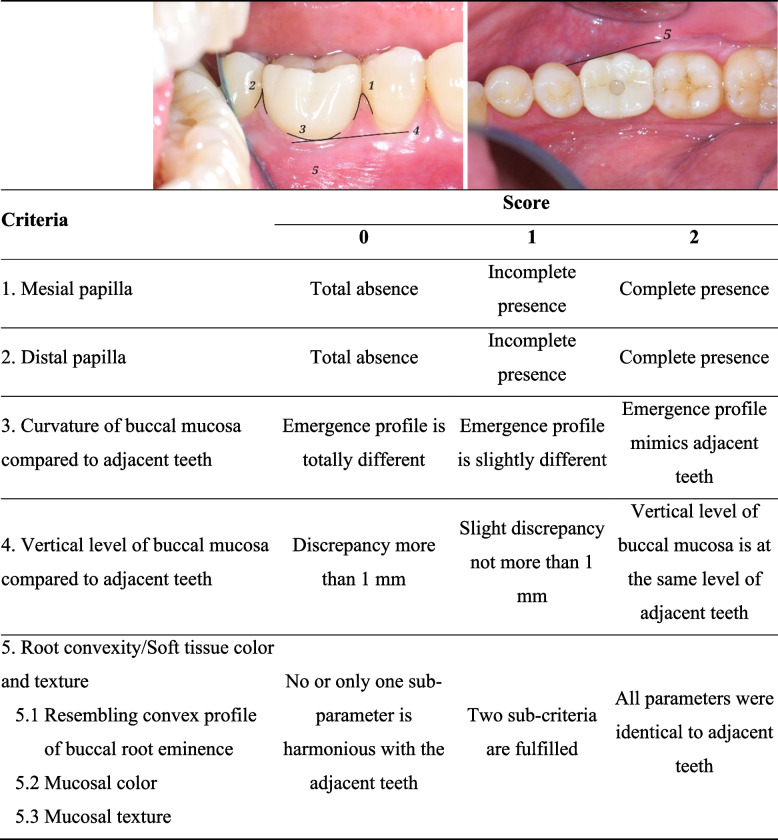
Modified Pink Esthetic Score (mPES) criteria [19]

**Table 6:** Survival and failure criteria, according to the health scale for dental implants [21]

**Categories**	**Criteria**
Success	a) No pain or tenderness upon function
	b) No mobility
	c) < 2 mm radiographic bone loss from initial surgery
	d) No exudate history
Satisfactory survival	a) No pain on function
	b) No mobility
	c) 2–4 mm radiographic bone loss
	d) No exudate history
Compromised survival	a) May have sensitivity on function
	b) No mobility
	c) Radiographic bone loss > 4 mm (less than 1/2 of implant body)
	d) Probing depth > 7 mm
	e) May have exudate history
Failure	Any of the following:
	a) Pain on function
	b) Mobility
	c) Radiographic bone loss > 1/2 length of implant
	d) Uncontrolled exudate
	e) No longer in mouth

### Statistical analysis

The data were expressed as mean, standard deviation, and percentage. Implant survival and success rates were calculated in percentages. The intra-examiner reliability was analyzed using intraclass correlation based on an absolute agreement, 2-way mixed-effects model [22], before measuring the marginal bone. The analysis was done using IBM SPSS statistics package version 21 (SPSS Inc, Chicago, IL, USA). As the distribution of marginal bone measurement was normal, the mean values of marginal bone on post-insertion prosthesis (T1) and follow-up (T2) radiographs were compared by a paired samples t-test with 95% confidence intervals. Patient satisfaction was reported through an average mean score.

## Results

### Patient demographic data

Out of 19 study subjects, there were 6 were men and 13 were women (mean age of 56 ± 15.03 years) ranging from 33 to 76 years (Table 1). Ten patients (52.63%) had underlying systematic diseases (ASA II category). Out of 25 implants, 18 were placed in the molar region and 7 were placed in the premolar region (10 were placed in the maxillary arch and 15 were in the mandibular arch) (Table 2). Seventeen implants were of the thick gingival biotype while eight implants were of the thin gingival biotype. Regarding the implant size, 10 implants of 4.8 × 10 mm size, 8 implants of 4.8 × 12 mm size, 6 implants of 4.1 × 12 mm size, and 1 implant of 4.1 × 10 mm size (Table 3). The implant restorations included 23 zirconia crowns and 2 full metal crowns.

### Patient satisfaction

The results of the patient satisfaction evaluated from 5 different categories are shown in Table 4. It shows that the average satisfaction rating for aesthetics was 4.75 ± 0.53, the function was 4.67 ± 0.64, the sense was 4.71 ± 0.46, the speech was 5, and the self-esteem was 4.71 ± 0.46. The average overall satisfaction score was 9.25 ± 0.90. Five patients reported food retention in the dental implant interproximal areas, which had to be removed using dental floss and/or interproximal brush.

### Clinical outcomes

#### Implant survival

A total of 24 implants (96%) survived and were in full function without infection at a median follow-up period of 57 ± 8.07 months (ranging from 42 to 67 months). One implant (4%) was explanted after 14 months of service.

#### Implant success

The results of the oral hygiene assessed by using an OHI-S index are shown in Table 7. It was found that 13 patients (68.42%) had good oral hygiene, and 6 patients (31.58%) had fair oral hygiene. The mean keratinized width of buccal maxillary mucosa was 6.63 ± 1.41 mm (range: 5–9 mm), buccal mandibular mucosa was 4.75 ± 2.15 mm (range: 2–9 mm), and lingual mandibular mucosa was 6.31 ± 1.74 mm (range: 3–9 mm) (Table 8).

**Table 7:** Results of oral hygiene evaluation from Oral Hygiene Index

Debris Index (DI-S)	Mean ± SD	0.74 ± 0.37
	Min–Max	0.17—1.33
Calculus Index (CI-S)	Mean ± SD	0.18 ± 0.31
	Min–Max	0.00—1.33
Oral Hygiene Index (OHI-S)	Mean ± SD	0.92 ± 0.55
	Min–Max	0.17—2.16
Summary of OHI-S (*n* = 19)		
• Good (n,%)	13, 68.42%	
• Fair (n,%)	6, 31.58%	

*SD* Standard deviation, *Min* Minimum, *Max* Maximum

**Table 8:** Results width of keratinized peri-implant soft tissue (mm)

Buccal maxillary soft tissue (*n* = 8)	Mean ± SD	6.63 ± 1.41
	Min–Max	5—9
Buccal mandibular soft tissue (*n* = 16)	Mean ± SD	4.75 ± 2.15
	Min–Max	2—9
Lingual mandibular soft tissue (*n* = 16)	Mean ± SD	6.31 ± 1.74
	Min–Max	3—9

*SD* Standard deviation, *Min* Minimum, *Max* Maximum

The implants that survived were assessed for marginal bone change. Marginal bone change as measured by one investigator from radiographs showed the intraclass correlation was 0.94 (95% confidence interval) which indicated excellent reliability. Twenty-four implants were classified as successful as the patients did not show any signs of pain or any other negative symptoms and were negative to percussion and palpation, with no mobility and suppuration. In general, the probing depths (measured at 6 points) were between 2 and 5 mm. Two implants had a 7 mm probing depth at one point. Hence, no signs of peri-implantitis were found. The mean mesial and distal marginal bone levels at T1 were 0.029 ± 0.575 mm and 0.009 ± 0.488 mm coronally to the implant platform, while the mesial and distal marginal bone levels at T2 were 0.165 ± 0.695 mm and 0.201 ± 0.717 mm. The mean differences of mesial and distal marginal bone change between T1 and T2 were 0.193 ± 0.874 (*p* = 0.290) and 0.210 ± 0.628 (*p* = 0.114), and were not significantly different (*P* > 0.05) (Table 9). The mean mPES score was 9.75 ± 0.44 (range: 9–10 score). The buccal contour was slightly different from the prosthetic insertion appointment which did not affect the esthetics (Fig. 4).

**Table 9:** Mesial and distal marginal bone level, compared to the shoulder of the dental implant at the time of prosthesis insertion and follow-up

	**Marginal bone level (mm)**		**Difference bone level (mm)**
	T1 (Mean ± SD)	T2 (Mean ± SD)	T2-T1 (Mean ± SD)
Mesial marginal bone	-0.029 ± 0.575	0.165 ± 0.695	0.193 ± 0.874 (*p* = 0.290)
Distal marginal bone	-0.009 ± 0.488	0.201 ± 0.717	0.210 ± 0.628 (*p* = 0.114)

(-) character means the marginal bone was coronal to the implant platform

*SD* Standard deviation, *T1* Post-insertion of the prosthesis, *T2* Follow-up visit

Twelve implants (50%) showed complications; 1 implant (4.17%) showed cement debonding, 2 implants (8.33%) showed screw loosening, and 10 implant crowns (41.67%) experienced proximal contact loss (floss could be passed through contact with little or no resistance), 5 implant crowns (20.83%) showed loss of material covering the screw hole, and 1 implant crown (4.17%), showed the attrition of material covering the screw. No biological complications were present and none of the implants showed any signs or symptoms of infection.

## Discussion

This prospective study analyzed the clinical outcomes of immediate implant placement in the posterior region that had survived a minimum of 3 years. Overall, patient satisfaction was high despite some food retention. The patients were able to clean the food-retentive area and maintain good oral hygiene. As this study was done on the posterior tooth areas, the satisfaction of the patients was dependent on comfort and function.

In our study, 96% of implants survived a follow-up period of 57 ± 8.07 months. This result was similar to previous studies [10, 11, 16]. A study by Mello et al. [10] demonstrated a 95.21% survival rate following immediate implant placement at least 6 months follow-up. Similarly, Lang [11] reported a 98.4% survival rate for 2 years suggesting that the patients who took post-operative antibiotics had a lower annual failure rate. A study by Ragucci et al. [16] also demonstrated a 96.6% survival rate of immediate implant placement in the molar tooth area after a 1-year follow-up. However, Meijer et al. [12] reported only a 73.3% survival rate. With proper implant dimensions, surface, design, and implant restoration, immediate implant placement in the posterior region with conventional loading can be performed with a high predictable success rate.

Risk factors for late implant failure can be categorized into 3 groups. Group 1 includes risk factors related to patient history. Group 2 includes the clinical parameters. Group 3 includes the technical factors [23]. The risk factors related to patients are the history of periodontitis, bruxism, radiotherapy, and early implant loss. Clinical risk factors are bone type 4 and posterior location. Smoking more than 10 cigarettes per day is the dominant risk for implant failure and in the presence of buccal dehiscence and/or infection, adds more risk [24]. Technical risk factors include low initial stability, > 1 implant placement during the surgery, and using the conus-type connection for implant-supported overdenture. In our study, ASA I and II patients were included for the immediate implant placement as both categories of patients bear similar risks for implant failure [25]. The systematic conditions that can affect the osseointegration of dental implants such as diabetes, cardiovascular disease, hypertension, and osteoporosis were excluded from our study [17]. Hence, the patient’s medical history had no bearing on the failure or success of the implant. Similarly, in this study, we choose fresh extraction sockets for the implant placement because the structure of sockets has slightly changed with age but it is not the same as the healed site that has a wide range of bone density variations.

In our study, 4% of the implants were failed. There are some predictions for implant failure in the fresh extraction socket. In a fresh extraction socket, the socket is broader compared to the delayed implant placement and the implants do not usually engage all the walls of the alveolar bone [26]. The primary implant stability can then be compromised as the implant is engaged only at the apical part of the socket [27]. However, in our study, there was adequate initial stability as measured by the ISQ monitor in all cases. The risk factor in this case might be an infection or improper occlusal loading, as the tooth was at the most distal location of the arch, bone type 4, or the presence of buccal dehiscence.

The keratinized mucosa width affects the peri-implant health in long-term stability as it decreases during the first 3 months but increases after 5 years [28]. The keratinized mucosa width of > 2 mm presents less gingival recession and periodontal attachment loss, compared to those with < 2 mm mucosa width. Moreover, higher plaque deposits, bleeding on probing, gingival inflammation, and gingival recession are associated with inadequate keratinized mucosa in implants [29]. In our study, all cases had keratinized mucosa width of > 2 mm suggesting that immediate implant placement in the posterior tooth area is successful at preserving keratinized tissue.

The bone graft also have important role in maintaining marginal bone of immediate implant placement [30, 31]. In our study, the marginal bone changes noted in the radiographs were not different which were different from the study by Bungthong et al. [32] where they found that the vertical bone height changed within 6 months after immediate implant placement as measured from the cone-beam computed tomography. However, bone remodeling is a continuous process and in the long term, the marginal bone changes may not be different. As the marginal bone on the radiograph was not corticated, it was difficult to define the border.

In this study, no biological complications were present, and none of the implants showed any signs or symptoms of infection. All of the successful implants were clinically and esthetically acceptable. The normal probing depth around the implant ranged from 2 to 6 mm [21]. In our study, 2 implants showed probing depths of 7 mm which can be because the error from the angulation of the probe next to the prosthesis contour might have affected the probing depth. It shows that immediate implant placement with customized healing abutment in the posterior tooth area maintains transmucosal tissue of the horizontal dimension [33], while horizontal buccal bone thickness reduces within the first 6 months after immediate implant placement [32]. In our study, following 3 years of implant placement, the bone remodeling continued altering the buccal dimension due to tooth function. However, the buccal soft tissue profile was preserved to promote self-cleansing of the implant area and did not affect patient satisfaction with esthetics.

Proximal contact loss between the natural teeth and implant-supported prosthesis was found in 10 out of 32 proximal contacts (31.25%) in our study. Greenstein and Varthis [34] reported that 34–66% of patients had proximal contact loss between the implant crown and natural teeth. The contact loss was more in the mandible than the maxilla and posterior teeth showed more than anterior teeth. Interproximal contact loss occurs as early as 3 months after prosthesis insertion [35], and the rate of contact loss increases as time passes appearing in the mesial rather than distal contact [36–40]. Proximal contact loss results in food impaction, increase in the caries rate, peri-implant mucositis, and peri-implantitis [37, 40–42]. The etiology of proximal contact loss is multifactorial. One study suggested recontouring the interproximal area of the adjacent tooth before the final impression to reduce the contact loss [34] and carefully assessing the distribution of occlusal force on the insertion appointment [43]. Occlusion and proximal contact of prosthesis should be re-evaluated regularly [35, 37, 40]. In our study, regular follow-up was done for all patients. It is important to maintain good oral hygiene and clean the proximal area of food impaction properly without infection.

Furthermore, 8.33% of implants in our study showed screw loosening. Brägger et al. [44] also reported 6.8% screw loosening after 4–5 years follow-up. The possible causes of screw loosening can be vibration, micromovement during functional loading, joint interface opening, and inadequate or loss of preload [45–47]. The fatigue character, friction, rotation at the implant–abutment interface, and component misfit can also affect preload [46, 47].

In our study, 4.17% of the crowns dislodged from the abutment which is similar to the previous study which found that the cement-retained implant prostheses showed a 4.7% loss of retention after a 5-year follow-up [48]. The dislodged crowns can be re-cemented with resin cement. The reason for cement debonding was unknown. However, it showed that convergent form, height of abutments, and permanent luting cement are important for the retention of implant prostheses [49]. Moreover, airborne abrasion of the abutment surface also improves the retention of the prosthesis [50, 51]. It is suggested to use a stronger adhesive cement in the cases of progressive loss of retention until enough retention is achieved [52]. The causes of cement debonding should be investigated in a future study. Further long-term studies can be done to study the implant success following immediate implant placement by increasing the implant sample size.

## Conclusions

Immediate implant placement in a posterior tooth with conventional loading yielded a predictable result. The implant survival rate was 96% at a mean time follow-up of 4 years and 9 months. The most prominent complications were proximal contact loss, followed by loosening of the screw and cement debonding. Oral hygiene and post-operative instruction were necessary for patient satisfaction and success. This research provides quantitative information for achieving better outcomes (success and survival) in immediate implants in posterior areas.

## Data Availability

The research data of this study can be requested from the corresponding author.
